# 3D magnetic resonance fingerprinting on a low-field 50 mT point-of-care system prototype: evaluation of muscle and lipid relaxation time mapping and comparison with standard techniques

**DOI:** 10.1007/s10334-023-01092-0

**Published:** 2023-05-18

**Authors:** Thomas O’Reilly, Peter Börnert, Hongyan Liu, Andrew Webb, Kirsten Koolstra

**Affiliations:** 1 Radiology, C.J. Gorter Center for MRI, Leiden University Medical Center, Albinusdreef 2, 2333 ZA Leiden, The Netherlands; 2 Radiology, Division of Image Processing, Leiden University Medical Center, Albinusdreef 2, 2333 ZA Leiden, The Netherlands; 3 Philips Research, Röntgenstraβe 24-26, 22335 Hamburg, Germany; 4 Computational Imaging Group for MR Diagnostics & Therapy, Center for Imaging Sciences, University Medical Center Utrecht, Heidelberglaan 100, 3584 CX Utrecht, The Netherlands

**Keywords:** Fingerprinting, Low field, Quantitative MRI, Halbach magnet, Matrix completion, ΔB_0_ map estimation

## Abstract

**Objective:**

To implement magnetic resonance fingerprinting (MRF) on a permanent magnet 50 mT low-field system deployable as a future point-of-care (POC) unit and explore the quality of the parameter maps.

**Materials and methods:**

3D MRF was implemented on a custom-built Halbach array using a slab-selective spoiled steady-state free precession sequence with 3D Cartesian readout. Undersampled scans were acquired with different MRF flip angle patterns and reconstructed using matrix completion and matched to the simulated dictionary, taking excitation profile and coil ringing into account. MRF relaxation times were compared to that of inversion recovery (IR) and multi-echo spin echo (MESE) experiments in phantom and in vivo. Furthermore, B_0_ inhomogeneities were encoded in the MRF sequence using an alternating TE pattern, and the estimated map was used to correct for image distortions in the MRF images using a model-based reconstruction.

**Results:**

Phantom relaxation times measured with an optimized MRF sequence for low field were in better agreement with reference techniques than for a standard MRF sequence. In vivo muscle relaxation times measured with MRF were longer than those obtained with an IR sequence (*T*_1_: 182 ± 21.5 vs 168 ± 9.89 ms) and with an MESE sequence (*T*_2_: 69.8 ± 19.7 vs 46.1 ± 9.65 ms). In vivo lipid MRF relaxation times were also longer compared with IR (*T*_1_: 165 ± 15.1 ms vs 127 ± 8.28 ms) and with MESE (*T*_2_: 160 ± 15.0 ms vs 124 ± 4.27 ms). Integrated ΔB_0_ estimation and correction resulted in parameter maps with reduced distortions.

**Discussion:**

It is possible to measure volumetric relaxation times with MRF at 2.5 × 2.5 × 3.0 mm^3^ resolution in a 13 min scan time on a 50 mT permanent magnet system. The measured MRF relaxation times are longer compared to those measured with reference techniques, especially for *T*_2_. This discrepancy can potentially be addressed by hardware, reconstruction and sequence design, but long-term reproducibility needs to be further improved.

**Supplementary Information:**

The online version contains supplementary material available at 10.1007/s10334-023-01092-0.

## Introduction

Low-field MRI (< 0.1 Tesla) has seen a resurgence in interest in recent years as a means of making MRI more affordable and accessible [1]. An obvious disadvantage of low-field MRI systems is the reduction in signal-to-noise ratio (SNR) compared to their high-field counterparts due to the supra-linear relationship between B_0_ field strength and SNR [2]. Some of the signal loss caused by the reduced magnetization and Larmor frequency can be recovered through efficient sequence design [3]; T_1_ times are significantly reduced at lower field strength [4, 5], T_2_ times are either the same or slightly longer [3, 6], and SAR is (ordinarily) not a limiting factor [7, 8]. One of the challenges with sequence optimization for low field is that while relaxation times are reported in healthy tissue, knowledge of tissue properties in the diseased state is less well known.

The reduction in system cost of a low-field MRI scanner has the potential to change the role of MRI in the healthcare system. MRI has been shown to be an effective method for screening for (early) disease, but the high cost associated with an MR examination typically limits the availability to people with an elevated risk of disease development [9–15]. The lower cost of low-field MR combined with increased implant safety [8] and reduced implant-induced image artefacts [8, 16] means that screening can be provided to a much larger fraction of the population. Efficient quantitative acquisition strategies may potentially play an important role in this [17, 18]. An additional advantage of quantitative methods is that they allow post-acquisition image synthesis of many different image contrasts using a single data set. This can lead to greater scan automation [19] and could potentially reduce the need for trained operators [1, 20]. Furthermore, it is possible to design low-field systems that are light weight and portable, making them deployable as point-of-care (POC) units [21].

Magnetic resonance fingerprinting (MRF) is a quantitative technique that can rapidly acquire multiple tissue parameters simultaneously [22]. The use of a variable flip angle pattern in a steady-state sequence results in unique signal evolutions for different tissue types, which can be quantified by matching the measured signal to a pre-calculated dictionary. The technique has been used in many clinical applications and has also potential for synthetic MR [23, 24]. However, so far most of the work on MRF has been performed on clinical scanners. Recently, Sarracanie et al. have shown that MRF is feasible at 100 mT using a fixed permanent magnet-based system with sufficient B_0_ homogeneity to enable balanced SSFP sequences to be run [25] and on a 6.5 mT system as well [26]. While susceptibility and chemical shift pose no significant issues at low field due to their scaling with B_0_ field strength and B_1_ distribution is unaffected by anatomy due to the much longer RF wavelength, other issues do arise. In particular, B_0_ homogeneity is often degraded [27, 28], especially in POC systems that are also susceptible to B_0_ field drift [29]. Furthermore, SNR is reduced because the lower B_0_ field strength, gradient strength and slew rate are reduced compared to standard clinical systems, and parallel imaging is typically not possible as only a single receive coil is used since there is little SNR to be gained by using coil arrays in the coil noise dominated regime [30, 31].

In this work, we implement 3D MRF on an in-house developed 50 mT permanent magnet-based low-field MRI system and explore the quality of the parameter maps. As a first application, we focus on muscle and lipid measurements of the lower limb, which has potential future applications in nutritional assessment in underserved communities. We use a Cartesian slab-selective spoiled steady-state with free precession (SSFP) sequence to reduce the sensitivity to B_0_ inhomogeneity and demonstrate that B_0_ induced image distortions can be corrected by adding ΔB_0_ encoding to the MRF sequence. We account for RF coil ringing, which distorts the RF pulse shape and has a stronger effect at low fields (due to the low sample loading effects and Larmor frequency) in the matching process and use a flip angle train optimized for the much shorter T_1_ times at 50 mT compared to 1.5 and 3 Tesla. We compare the matched relaxation times to those obtained with reference techniques in phantom and in vivo experiments. Finally, we use the obtained MRF relaxation times to synthesize several MRI contrasts retrospectively, to test the feasibility of simplifying the workflow of low-field MRI systems through a one-scan-only approach.

## Methods

### Hardware

**Fig. 1:**
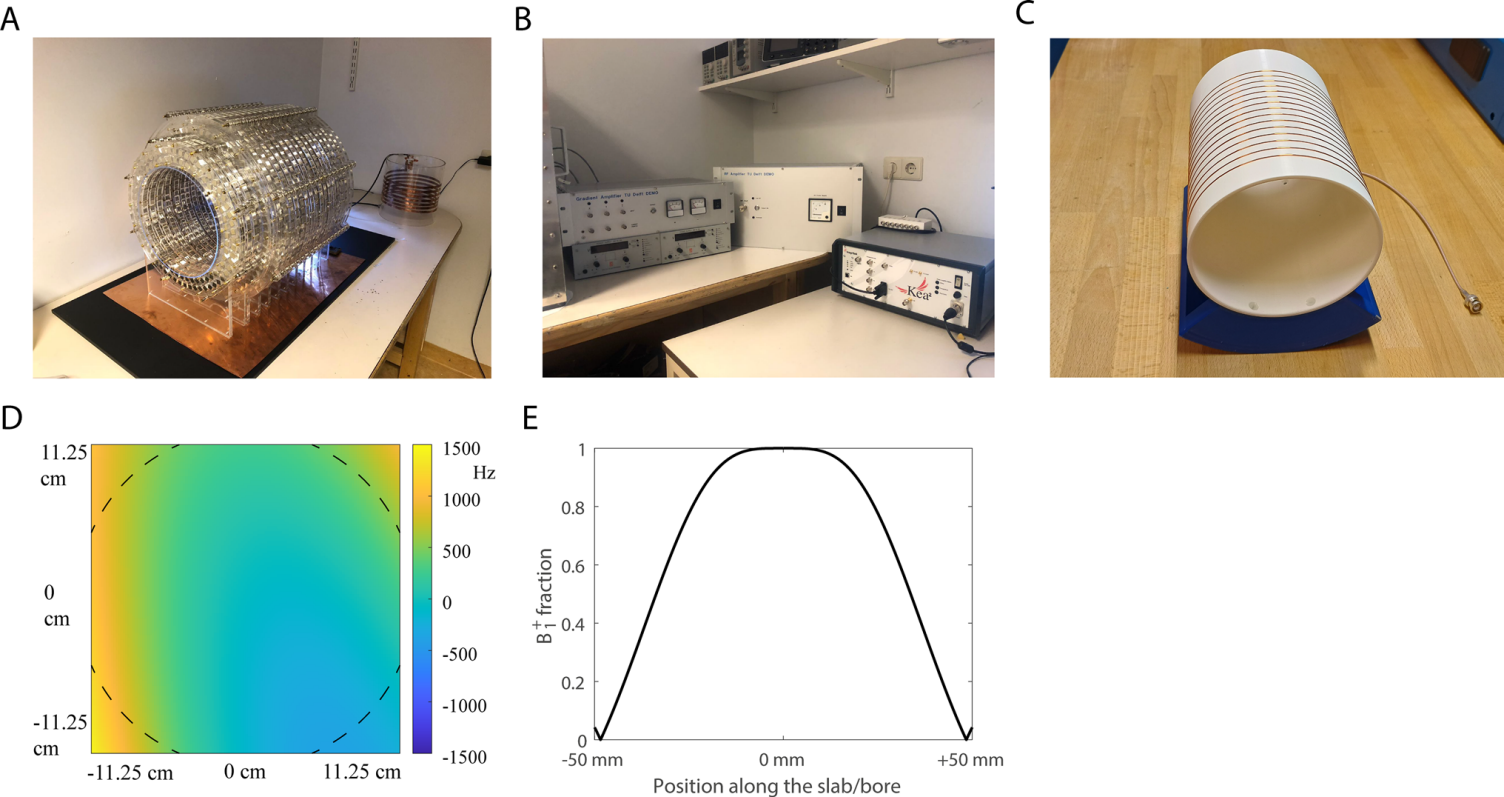
Experimental setup. **A** The Halbach magnet consists of 23 rings filled with neodymium boron iron magnets. **B** The gradient amplifiers (left), RF amplifier (middle) and the spectrometer (right). **C** An RF solenoid coil was used for transmitting and receiving the signal. **D** The main magnetic field was measured with a robot. Values are shown as difference in Hz with respect to the center frequency. **E** The simulated B_1_^+^ profile along the main axis of the solenoid

All data were acquired on an in-house developed 50 mT Halbach-based MRI scanner described in detail previously [27, 32], shown in Fig. 1. The total weight of this system is about 100 kg (magnet: ~ 70 kg, gradients: ~ 7 kg, amplifiers: ~ 20 kg). Typical linewidths, measured as the full width at half maximum of a spectrum, were around 100 Hz on the lower leg after applying linear shims using the gradient coils. Heat introduced by the body causes an f_0_ drift of around 1300 Hz per hour. Therefore, f_0_ was determined prior to every scan to minimize RF off-resonance effects. The heating of the magnet is spatially homogenous, and the line width does not significantly broaden between the beginning and end of the scan; we therefore perform shimming only at the start of the scan session.

A 15 cm-long, 15 cm-diameter solenoid coil with 15 turns of 0.8 mm copper wire is used for RF transmit and receive. The loaded Q factor of the coil is around 70 giving a coil bandwidth of 30 kHz, sufficient to avoid any coil bandwidth-related image shading. The RF pulse from the spectrometer is amplified by a custom-built 1 kW RF amplifier described previously [29]. Power optimization is performed at the start of every imaging experiment by recording a series of FIDs with increasing power and integrating underneath the resulting spectra. A sinusoidal function is fitted to the integrated spectra to determine the power needed for a 90° and 180° flip angle. The power optimization is performed separately for the 100 μs excitation and the 200 μs inversion pulses due to the nonlinear scaling caused by ringing of the RF coil. The rise and decay time of the RF pulse was around 16 μs and is considered in the slab profile correction in the matching process. An RF shielding blanket was placed over the subject to minimize external noise coupling into the RF coil.

### Fingerprinting implementation

**Fig. 2:**
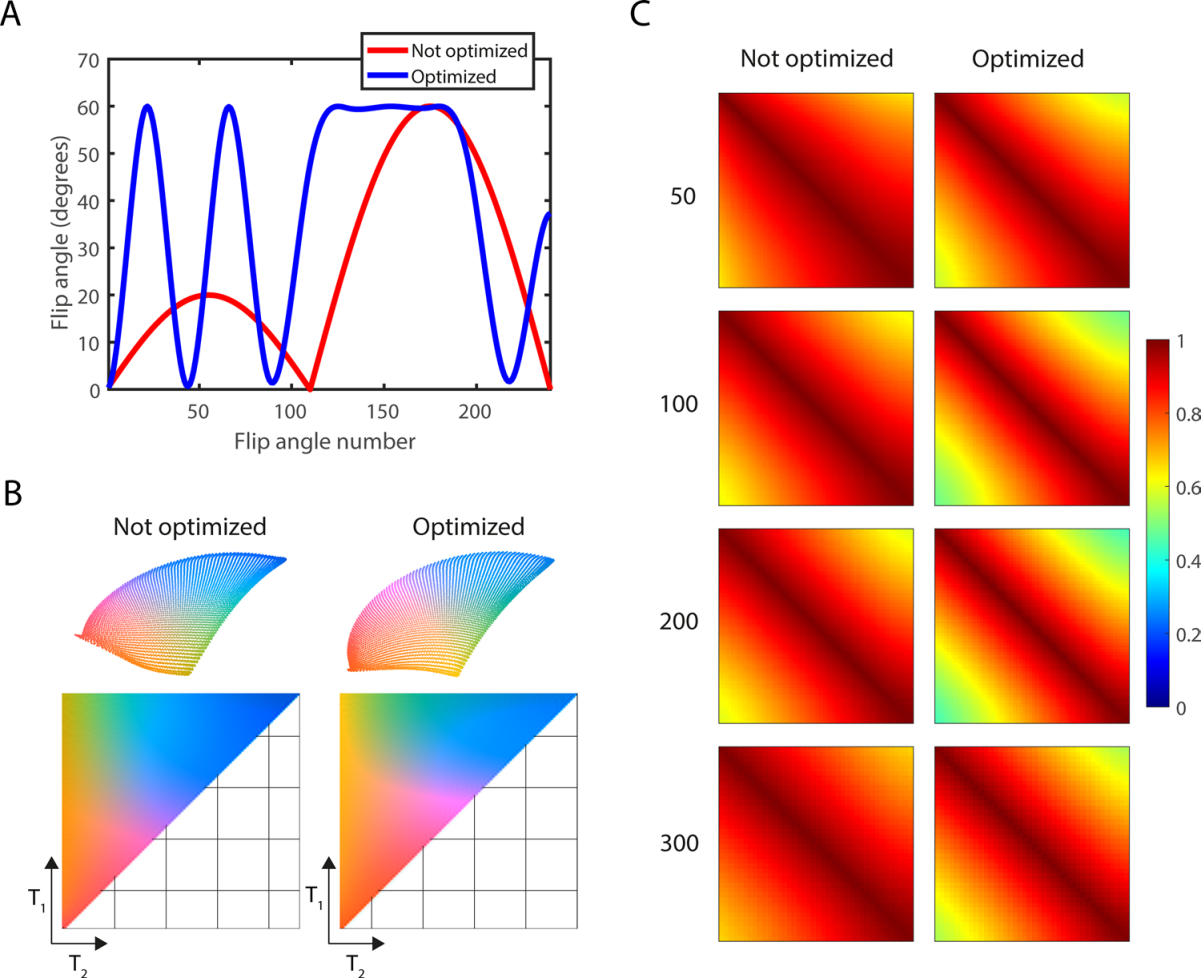
MRF flip angle pattern and k-space sampling pattern. **A** The optimized flip angle pattern (blue) and standard flip angle pattern (red) used for the experiments. **B** The three-dimensional embeddings of the corresponding dictionaries obtained with t-SNE (top) and the corresponding color-coded dictionary maps in *T*_1_/*T*_2_ space (bottom). **C** The similarity maps for fixed *T*_2_ values (shown on the left) show a steeper diagonal structure for the optimized sequence compared to the standard sequence, confirming improved encoding capability. Similarity maps are shown for the *T*_1_ ranges corresponding to each fixed *T*_2_, i.e., from top to bottom: *T*_2_ = 50: *T*_1_ = 55–500, *T*_2_ = 100: *T*_1_ = 105–500, *T*_2_ = 200: T_1_ = 205–500, *T*_2_ = 300: *T*_1_ = 305:500

MRF data were acquired with a (spoiled) FISP sequence [33, 34], using an unbalanced gradient in the readout direction implemented on a Magritek Kea2 (Aachen, Germany) spectrometer. The other two phase encoding gradients were implemented in balanced mode, and no RF spoiling was used. An MRF flip angle train was designed using Cramér–Rao-bound optimization for optimal discrimination between four different (T_1_,T_2_) pairs: (247,85), (184,48), (122,85), (89,90) ms corresponding to the phantom described in the next section, and to five (*T*_1_,*T*_2_) pairs for the in vivo experiments: (20,20), (42,42), (120,80), (160,160), (440, 440) ms. In this process, the number of flip angles was fixed to 240, TR fixed to 12 ms, and the maximum flip angle was restricted to 60°. An unoptimized reference sequence based on the one used in Ref. [34] used the same number of shots, TR and maximum flip angle. The patterns are shown in Fig. 2A. TE was set to constant values of 6 ms. These two flip angle patterns and the corresponding dictionaries were analyzed with t-SNE, using the method described in Ref. [35], to confirm that the encoding capability was higher for the optimized sequence compared to the non-optimized sequence, as shown in Fig. 2B, C. The inversion pulse length was set to 100 µs, to ensure sufficient inversion efficiency for the entire field of view (FOV). A 200 µs block pulse was used to excite a 3D imaging volume during the MRF train with a slab selective gradient of 100 kHz/m applied in the head/foot direction along the main axis of the magnet. The extended phase graph formalism was used to compute a three-dimensional dictionary [36, 37] for different *T*_1_/*T*_2_/*B*_1_^+^ combinations, containing a total of 145,500 elements. *T*_1_ values ranged from 20 to 500 ms in steps of 5 ms, and T_2_ values ranged from 10 to 500 ms in steps of 5 ms. A B_1_^+^ fraction (defined as the fraction of the measured value from the pulse calibration) ranging from 0.05 to 1.50 in steps of 0.05 was incorporated into the dictionary calculation to support slab excitation profile correction. The relatively short T_1_ times at 50 mT allowed us to set the time between repetitions of the MRF train to 0.5 s. For the relatively longer *T*_1_ values (*T*_1_ ~ 250 ms), a steady state was reached after a single MRF train. This train contributed to less than 1% of the total signal for the adopted 3D Cartesian readout, such that any remaining steady-state effects on the matched parameter maps can be ignored.

### Phantom construction

The phantom was constructed out of 110-mm-diameter, 80-mm-long plexiglass tube, with 3 additional 30-mm-diameter plexiglass tubes placed inside. The main tube structure was filled with a ‘reference liquid’ (*T*1 = *T*2 =  ~ 90 ms); the smaller tubes were filled with tissue mimicking liquids made from a mixture of agarose, copper sulfate and water with the following concentrations: (1) white matter: 1.6 mmol/L CuSO4, 1% agarose by mass, (2) muscle: 2 mmol/L CuSO4, 2% agarose by mass (3) lipid: 4 mmol/L CuSO4, 0.5% agarose by mass, (4) background: 6 mmol/L CuSO4, no agarose. This resulted in (*T*_1_,*T*_2_) combinations corresponding to (1) white matter: T_1_/T_2_ = 247/85 ms, (2) muscle: *T*_1_/*T*_2_ = 184/48 ms, (3) lipid: *T*_1_/*T*_2_ = 122/85 ms and (4) background: *T*_1_/*T*_2_ = 89/90 ms. These T_1_/T_2_ values were confirmed with spectroscopic measurements.

### MR Data acquisition

**Fig. 3:**
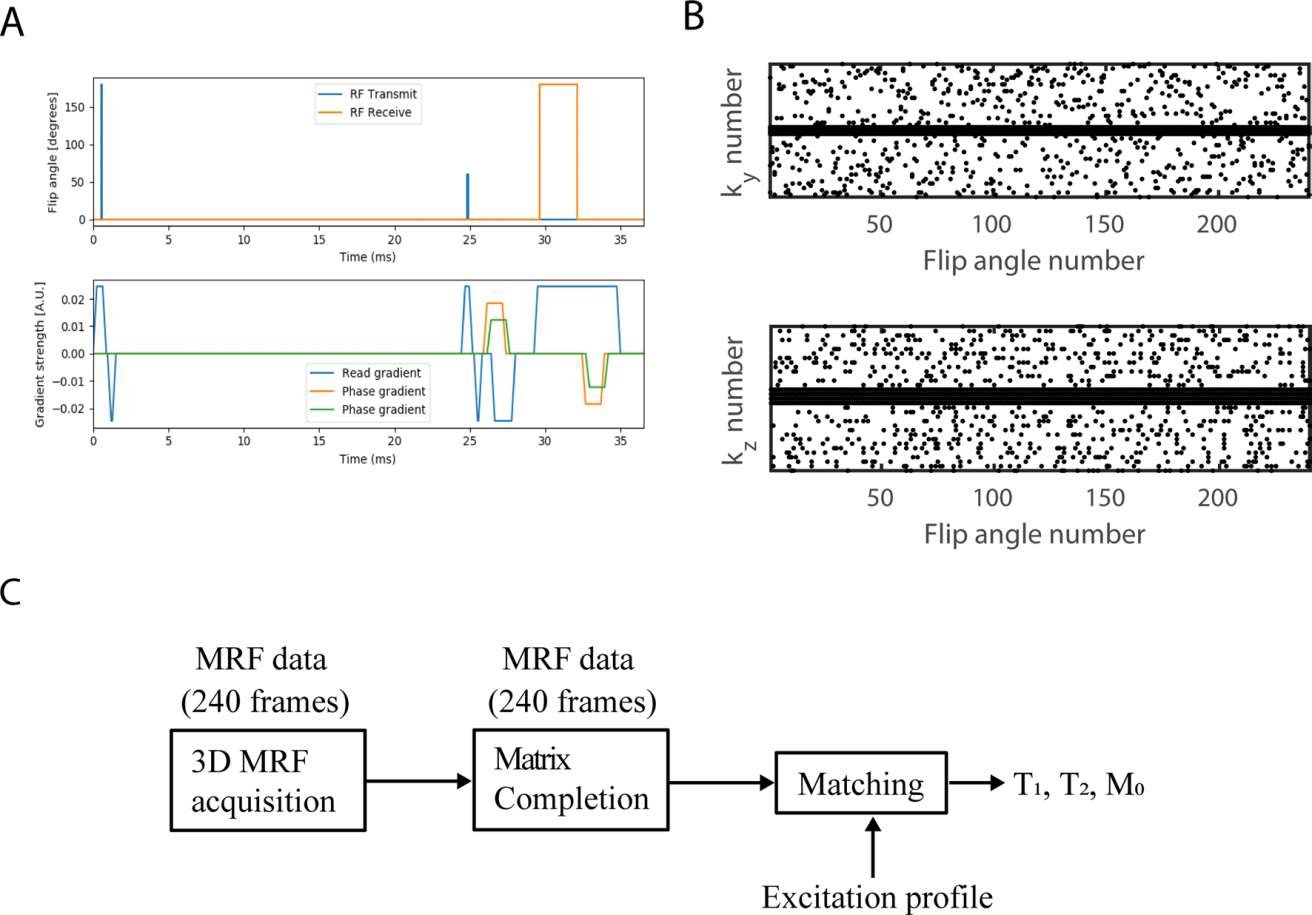
**A** Schematic overview of the MRF pulse sequence. This includes the time from the start of the inversion pulse until the end of the first TR. This is a spoiled slab-selective 3D SSFP sequence, with unbalanced gradients in the readout direction. **B** 4D sampling scheme. For each MRF frame, the c × c × n_readout_ center region of k-space was always acquired and used to estimate the rank of the MRF data. Note that the readout dimension (not shown) was always fully sampled. **C** Processing pipeline for the MRF data

Experiments were performed in a phantom and in 7 healthy volunteers after informed consent was obtained, conforming to the local ethical regulations. The data from one volunteer were corrupted and was not included in the analysis. MRF scans were acquired with a Cartesian sampling scheme in undersampled mode, acquiring a 4 × 4 fully sampled center region, while acquiring the rest of k-space in a random fashion, according to the sampling scheme shown in Fig. 3B.

Phantom scans used the following parameters: FOV = 150 × 150 × 100 mm^3^, resolution = 2.5 × 2.5 × 5.0 mm^3^, TE/TR = 6/12 ms, imaging bandwidth (BW) = 333 Hz/pixel, total undersampling factor = 7.5, scan time = 9 min. In vivo scans used the following scan parameters: FOV = 170 × 150 × 99 mm^3^, resolution = 2.5 × 2.5 × 3.0 mm^3^, TE/TR = 6/12 ms, imaging bandwidth (BW) = 294 Hz/pixel, total undersampling factor = 8.6, scan time = 13 min and 6 s. In the phantom experiment, the MRF scan was acquired with the optimized flip angle pattern as well as with a more standard flip angle pattern used at high field in Ref. (38) for comparison. Spectroscopic reference measurements were obtained in each tube individually using six inversion times (25/50/75/100/150/300 ms) for *T*_1_ values and Carr–Purcell–Meiboom–Gill (echo times 20/40/60/80/100/120/140/160/180/200 ms) for T_2_ values. In one of the volunteers and in the phantom, standard quantitative (imaging) techniques were used to produce reference relaxation time maps on the same FOV as the MRF scans. Inversion recovery (IR) was used for *T*_1_ mapping: resolution = 2.5 × 2.5 × 8.3 mm^3^, TE/TR = 12/900 ms, echo train length (ETL) = 5, inversion times = 25/50/75/100/150/300 ms, imaging bandwidth (BW) = 294 Hz/pixel, scan time = 12 min and 58 s. Multi-echo-spin-echo (MESE) was used for *T*_2_ mapping: resolution = 2.5 × 2.5 × 8.3 mm^3^, TR = 1250 ms, TEs = 20/40/60/80/100/120/140/160/180/200 ms, imaging bandwidth (BW) = 294 Hz/pixel, scan time = 15 min. In the phantom experiment, reference techniques were acquired on the same resolution as the MRF scans. Finally, in one of the volunteers, an alternating TE pattern (ΔTE = 150 µs) was implemented along the MRF train to support MRF-integrated ΔB_0_ estimation. A TSE-based B_0_ map [39] was acquired on the same FOV for comparison using the following scan parameters: resolution = 2.5 × 2.2 × 3.0 mm^3^, TE/TR = 12/300 ms, readout gradient shift = 150 µs, echo train length (ETL) = 5, imaging bandwidth (BW) = 294 Hz/pixel, scan time = 9 min.

### Reconstruction of MRF images

Full MRF k-space data were reconstructed from undersampled k-space data using a matrix completion-based reconstruction [38, 40]. The fully sampled lines in the 4 × 4 center of the 3D k-space matrix were used as calibration data. The 5 most significant singular values obtained with the singular value decomposition (SVD) were used to estimate the rank of the MRF data and to form a projection matrix; 50 Iterations were used to ensure convergence. After reconstruction, the 3D k-space data were filtered using a 3D sine-bell squared filter to reduce noise.

For one of the volunteers, a ΔB_0_ map was estimated from the alternating TE pattern (TE_1_ = 6 ms, TE_2_ = 6.15 ms) in the MRF sequence, as shown in Online Resource 3A. This was done by first reconstructing the time series corresponding to each TE with matrix completion. After this, two averaged images were computed for TE_1_ and TE_2_, containing a phase difference introduced by B_0_, from which ΔB_0_ was reconstructed using total variation regularization and spherical harmonic decomposition. This ΔB_0_ map was used in a model-based reconstruction framework to correct for image distortions [39] after which the combined MRF images were matched to the original dictionary. This processing pipeline is schematically shown in Online Resource 3B, where the gray boxes indicate the additional processing steps that are performed for the MRF data acquired with the alternating TE pattern.

### Matching process

The shape of the RF pulse in the time domain was measured using an oscilloscope connected to a pickup coil inside the transmit coil. The corresponding excitation profile was calculated using the Shinnar–Le–Roux (SLR) algorithm, resulting in an inhomogeneous excitation profile along the bore, as plotted in Fig. 1E. This excitation profile was considered in the matching process as follows. For each slice, a sub-dictionary was selected containing only the dictionary elements with an excitation fraction (B_1_^+^ fraction between 0.05 and 1.50) equal to that of the corresponding slice. Second, each slice was matched to its own sub-dictionary. B_1_^+^ variations due to wave interference were ignored, since these have a minimal effect for the solenoid RF coil used at 50 mT. The matching was performed in 3D after normalizing the dictionary entries, using the inner product as similarity measure. A schematic overview of the processing pipeline can be found in Fig. 3C.

### Synthetic MRI

The matched T_1_ and T_2_ maps obtained with MRF were used to compute synthetic MRI images corresponding to the IR and MESE sequences. This was done by substituting the matched T_1_ and T_2_ maps in the signal equations.

$${S}_{IR}\left(TI\right)={M}_{0} \left(1-2{e}^{\frac{TI}{{T}_{1}}}+{e}^{-\frac{TR}{{T}_{1}}}\right) \mathrm{ \, and \, }{S}_{MESE}\left(TE\right)={M}_{0}{e}^{-\frac{TE}{{T}_{2}}}$$

using the TI, TR and TE settings that were used for the IR and MESE measurements performed in this study. This allowed a comparison between MRF and standard quantitative measures in terms of generated synthetic images. These synthetic images show the effect of over-/underestimation of relaxation times on the MR image contrast and therefore put the accuracy of the matched MRF times into perspective.

Reconstruction and matching of the MRF data were performed in MATLAB (Mathworks Inc, Natick, MA) and run on a Windows 64-bit machine with Intel Xeon CPU E5-1620 v3 @3.5 GHz and 32 GB internal memory. The processing time for the volumetric MRF data was 29 s for matrix completion reconstruction and 6 min for matching.

## Results

**Fig. 4:**
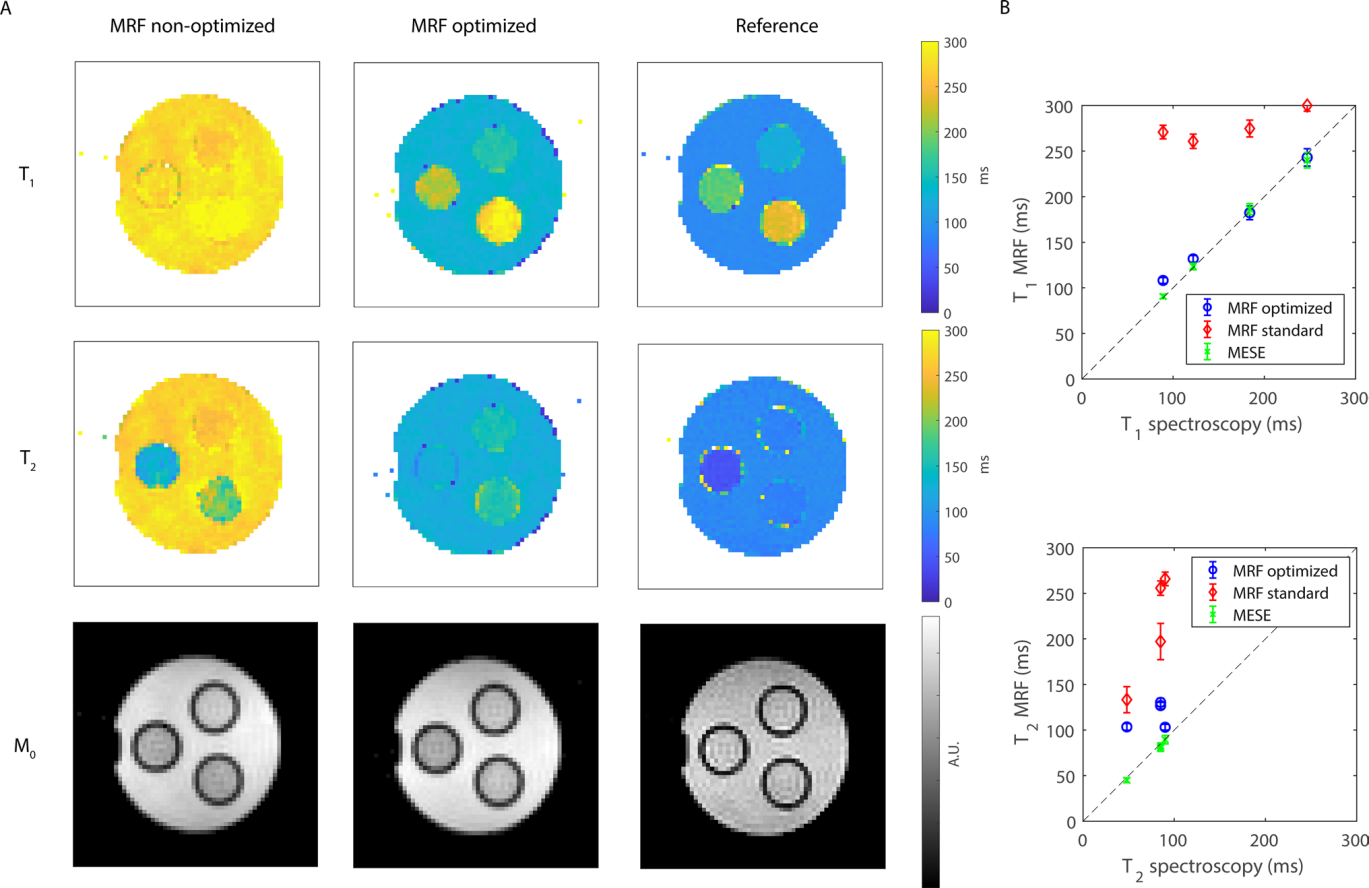
Relaxation time maps in a phantom: comparison between MRF and reference techniques. **A** The *T*_1_, *T*_2_ and *M*_0_ maps obtained with the optimized flip angle pattern contain less noise compared to those obtained with the standard flip angle pattern. **B** The *T*_1_ and *T*_2_ values obtained with the optimized sequence are much closer to the *T*_1_ and *T*_2_ values obtained with the reference techniques (IR and MESE) and to spectroscopic measurements, compared with those obtained with the standard flip angle pattern. The error bars represent standard deviations computed in each of the ROIs. The standard deviation of the spectroscopic measurements (computed as the standard deviation over the residuals of the fit) was less than 1% for each sample measured individually, which is much lower than that obtained with the imaging sequences (standard and MRF)

Figure 4 shows the *T*_1_, *T*_2_ and *M*_0_ maps measured with the optimized and the non-optimized flip angle patterns in a phantom compared to the reference techniques (IR and MESE) and compared to spectroscopic measurements. The maps acquired with the optimized flip angle pattern contain much less noise compared to those measured with the non-optimized one, and the relaxation time values are much closer to those measured with the reference techniques. This is confirmed by the numbers plotted in Fig. 4B, showing the relaxation times averaged in an ROI for each of the four phantom compartments, with a maximum *T*_1_/*T*_2_ difference of 19/55 ms. Note that the noise-like pattern in the parameter maps (hereafter referred to as noise) is not noise in the traditional sense since it is a result of many influencing factors, including the discretized dictionary matching, the low SNR of the underlying MRF signals and the undersampling effects superimposed in those pixels, that might impair perfect pattern matching. The standard deviation of the spectroscopic measurements (computed as the standard deviation over the residuals of the fit) was less than 1% for each sample measured individually, which is much lower than that obtained with the imaging sequences (standard and MRF). Online Resource 1 shows that the adopted acquisition/reconstruction scheme is robust to undersampling: undersampling with a factor of 7.5 results in negligible relaxation time differences.

**Fig. 5:**
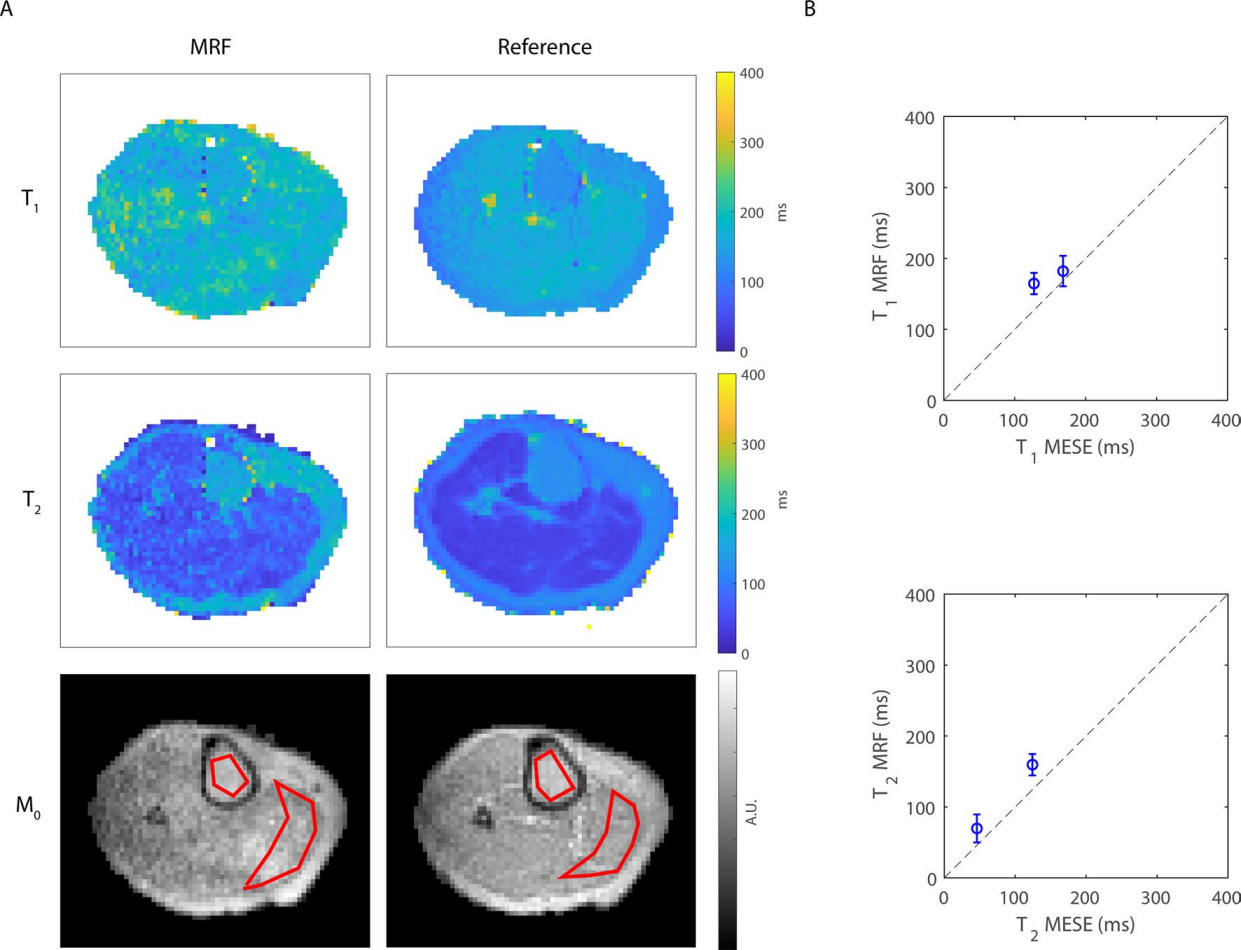
Relaxation time maps in a healthy volunteer: comparison between MRF and reference techniques. *T*_1_, *T*_2_ and *M*_0_ maps obtained with MRF are in the same range as those obtained with an IR sequence (182 ± 21.5 ms vs 168 ± 9.89 ms), but the muscle *T*_2_ times measured with MRF are longer than those measured with an MESE sequence (69.8 ± 19.7 ms vs 46.1 ± 9.65 ms). The maps obtained with the reference techniques contain less noise compared to those obtained with MRF due to larger voxel size and TSE-based sequences. ROIs used to compute mean and standard deviations in the bone marrow and the gastrocnemius muscle are shown in red in the M_0_ maps. **B** The *T*_1_ and *T*_2_ values averaged over an ROI in the muscle and in the bone marrow are overestimated compared to the reference techniques (IR and MESE), potentially influenced by the lower SNR in the MRF scans. Note that the field drift during the undersampled in vivo MRF scans was about 200 Hz. The error bars represent standard deviations computed in each of the ROIs

Figure 5 shows a comparison between the optimized MRF sequence and the reference techniques in the center slice of one volunteer’s calf. The muscle *T*_1_ and *T*_2_ times measured with MRF are longer than those measured with an IR sequence (*T*_1_: 182 ± 21.5 ms vs 168 ± 9.89 ms) and with MESE (*T*_2_: 69.8 ± 19.7 ms vs 46.1 ± 9.65 ms), respectively. In the bone marrow, *T*_1_ and *T*_2_ times are also longer with MRF compared with IR (*T*_1_: 165 ± 15.1 ms vs 127 ± 8.28 ms) and with MESE (*T*_2_: 160 ± 15.0 ms vs 124 ± 4.27 ms). The noise in the MRF maps is higher, due to the higher resolution of MRF (2.5 × 2.5 × 3.0 mm^3^) compared to reference techniques (2.5 × 2.5 × 8.3 mm^3^), the high undersampling factor for MRF (*R* = 8.6) compared to reference techniques (*R* = 1) and the different type of sequences used (gradient echo vs spin echo).

**Fig. 6:**
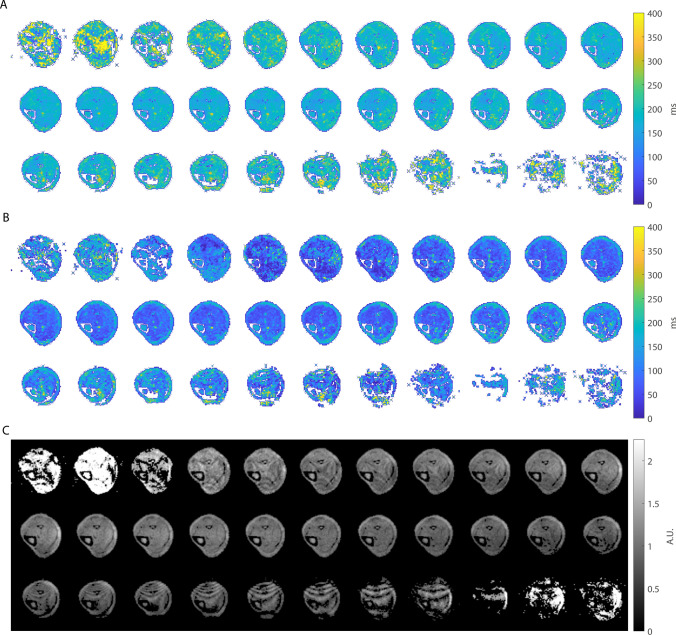
Volumetric relaxation time maps in one healthy volunteer. The *T*_1_ (**A**), *T*_2_ (**B**) and *M*_0_ (**C**) maps after excitation profile correction show consistent values along the slice dimension. The slices toward the edges of the excited region show more noise due to lower maximum flip angles used as well as a lower receive coil sensitivity in these regions

The volumetric MRF maps along the entire imaging stack for another volunteer are shown in Fig. 6. The estimated *T*_1_ and *T*_2_ values are consistent along the bore in the volume of the RF coil where the B_1_^+^ is relatively homogeneous, with the noise amplitude increasing toward the edges of the excited volume as expected. Online Resource 2 presents the same maps, but without excitation profile correction, showing increasing apparent *T*_2_ values and decreasing apparent M_0_ values away from the center slice, thus showing the importance of excitation profile correction. Table 1 summarizes the MRF relaxation times (mean ± standard deviation) measured in an ROI in the muscle and in the bone marrow in each of the 6 volunteers, with overall mean *T*_1_ and *T*_2_ values of 182 ms and 66.2 ms in the muscle and 160 ms and 149 ms in the bone marrow.

**Table 1:** Relaxation times in 6 volunteers

Vol. #	Muscle	Bone marrow
**T**_**1**_ (ms)		
1	191 ± 27.4	161 ± 26.4
2	157 ± 15.0	168 ± 14.3
3	177 ± 24.6	147 ± 16.9
4	185 ± 19.8	165 ± 18.6
5	182 ± 21.5	165 ± 15.1
6	200 ± 35.3	155 ± 24.8
Mean ± std	182 ± 14.6	160 ± 7.89
**T**_**2**_ (ms)		
1	69.6 ± 29.4	131 ± 39.7
2	61.9 ± 12.4	159 ± 17.3
3	52.5 ± 15.9	141 ± 17.9
4	70.9 ± 17.5	152 ± 28.0
5	69.8 ± 19.7	160 ± 15.0
6	72.3 ± 33.4	150 ± 25.2
Mean ± std	66.2 ± 7.63	149 ± 11.0

Mean ± standard deviation values (ms) in a region of interest are reported for the muscle and the bone marrow

**Fig. 7:**
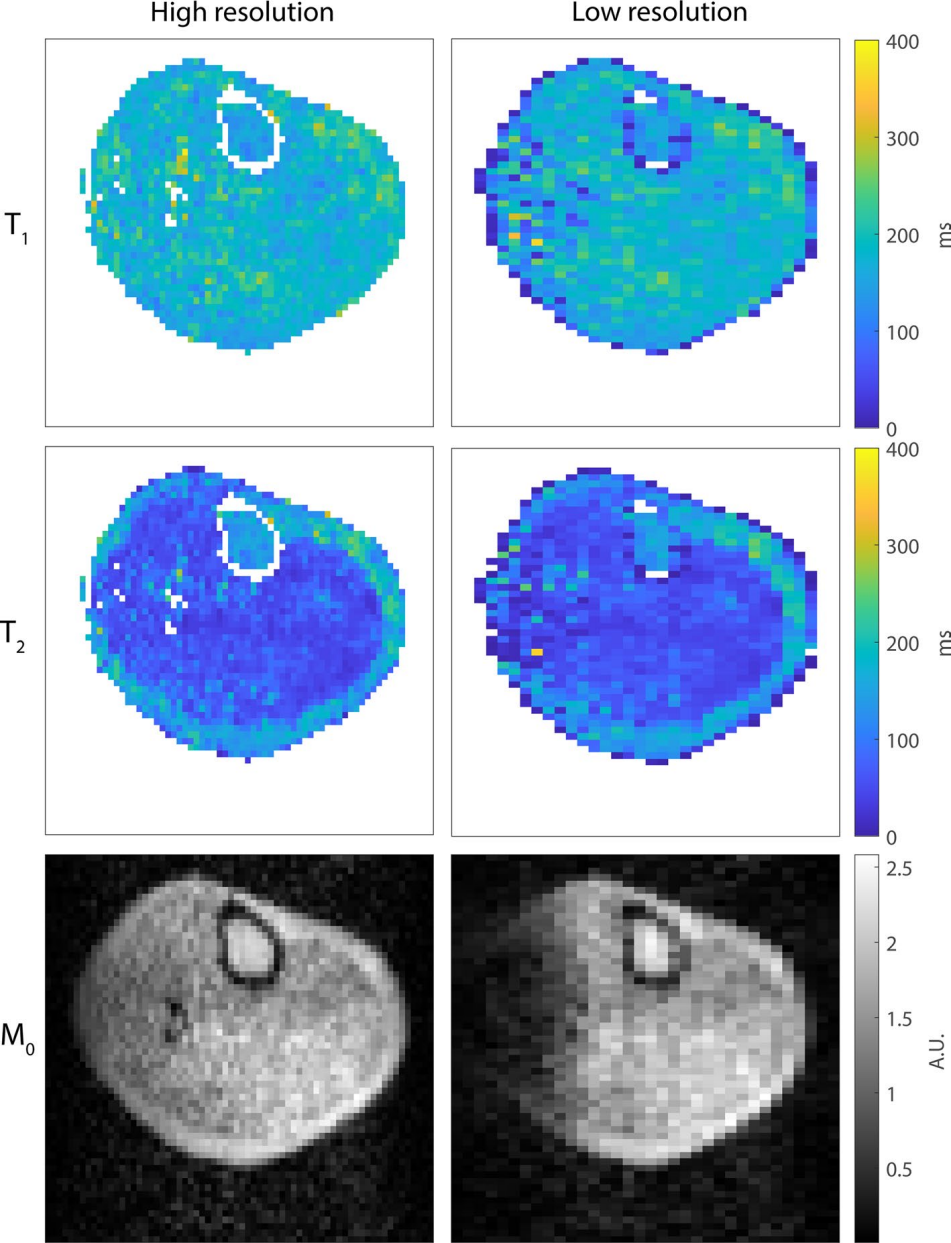
Effect of intra-voxel dephasing on the quality of the MRF parameter maps. The *M*_0_ map matched from the low-resolution (2.5 × 5.0 × 3.0 mm^3^) MRF data shows a large area with reduced signal at the left side of the calf due to intra-voxel dephasing. This results in more noise in the corresponding regions in the *T*_1_ and *T*_2_ maps compared to the high-resolution maps (2.5 × 2.5 × 3.0 mm^3^)

One way to reduce the total MRF scan time is by reducing the spatial resolution of the MRF data. However, in inhomogeneous B_0_ fields this leads to increased intra-voxel dephasing. Figure 7 shows this effect in one volunteer: reconstructing the same data with a twice as coarse a resolution in the readout dimension results in a large reduction in signal in the left part of the calf, where the B_0_ inhomogeneities are strongest. This can be observed in the *M*_0_ map and leads to a larger amount of noise in the corresponding regions in the *T*_1_ and the *T*_2_ maps.

Using an alternating TE pattern along the MRF train results in very similar parameter maps compared to using a constant TE, as shown in Online Resource 3. Estimating a ΔB_0_ map from the alternating TE pattern and using it in a model-based MRF reconstruction result in matched parameter maps with slightly reduced distortions. This effect would be more pronounced in case of a stronger inhomogeneous ΔB_0_ field. This experiment also shows that the estimated MRF ΔB_0_ map is in the same range as that estimated from a TSE sequence, with a maximum error of 105 Hz.

**Fig. 8:**
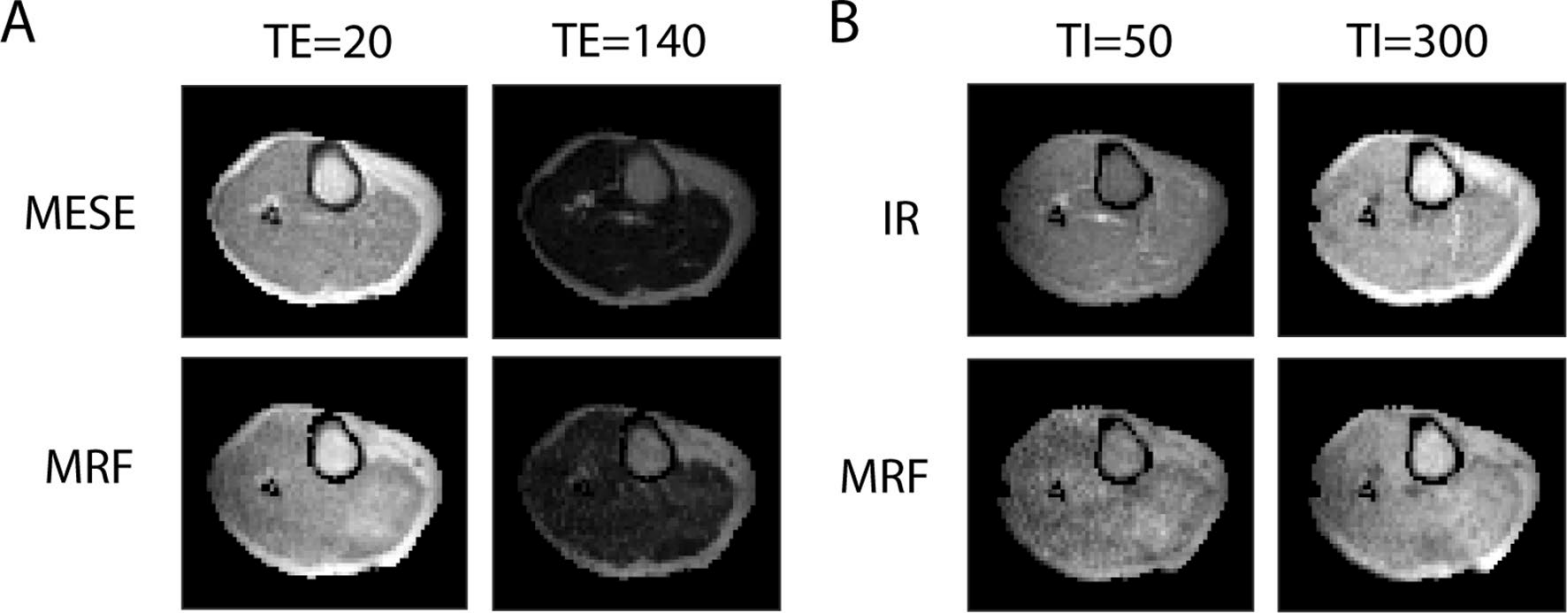
Synthetic MRI images of a volunteer’s lower leg obtained with MRF and with reference relaxation time maps. MRF data were first averaged over three neighboring slices before matching to the dictionary to obtain relaxation time maps with a similar voxel size (2.5 × 2.5 × 9.0 mm^3^) as the reference maps (2.5 × 2.5 × 8.3 mm^3^). **A** T_2_ maps measured with MESE and with MRF were used to simulate TSE images at echo times TE = 20 ms and TE = 140 ms. **B** *T*_1_ maps measured with IR and with MRF were used to simulate IR TSE images at different inversion times TI = 50 ms and TI = 300 ms. Note that the parameter matching step served as a noise filtering step in this process. A comparison with the source reference data can be found in Online Resource 4

Figure 8 shows that the MRF relaxation time maps can be used to generate synthetic MRI images, in which the MRF parameter matching step effectively serves as a noise filtering step. Online Resource 4 shows a comparison with all the source data, also demonstrating the effect of the overestimation of MRF relaxation times on the synthetic data.

## Discussion

In this work, we implemented MRF on a custom-built 50 mT permanent magnet system. We used a three-dimensional Cartesian readout with two-dimensional undersampling (*R* = 7.5–8.6) and a matrix completion-based reconstruction to obtain volumetric parameter maps. We showed the proof-of-principle for a volumetric multi-parametric scan with a total acquisition time of ~ 13 min. The muscle *T*_1_ times measured in vivo with MRF were slightly longer compared to those measured with an IR sequence (8.3% difference), while the estimated muscle *T*_2_ times were much longer with MRF compared to MESE (51.4% difference). In the bone marrow, both the *T*_1_ and the *T*_2_ values were overestimated compared to IR (30%) and MESE (29%). Estimating a ΔB_0_ map from the MRF data using an alternating TE pattern and using it in a model-based reconstruction framework resulted in parameter maps with reduced geometric distortions.

A large overestimation of T_2_ values was consistently observed in muscle tissue in all healthy volunteers with respect to reference measurements in this study and compared to the literature [41]. Fully explaining relaxation time differences between MRF and other techniques has traditionally been challenging, and much previous published work has concentrated on this effort. For example, Ref. [34] found an underestimation of white matter *T*_2_ (17.7%) while gray matter T_2_ measures were within the literature range in the same subjects. Muscle MRF T_2_ values were overestimated compared to reference techniques, which was in part explained by the presence of fat and flow [42, 43], but even after taking these into consideration a considerable discrepancy remained (37%). Correcting for B_1_^+^ and slice profile effects improved parameter quantification in phantoms, but generally still resulted in underestimation of *T*_2_ values compared to the literature in vivo (gray matter: ~ 43%, white matter: 41%) [44].

Accurate and reproducible relaxation time measurements using MR fingerprinting are particularly challenging on a custom-built low-field system such as used here. In addition to the intrinsic low SNR, many of the electronic components have been designed with total cost being an important criterion. This means that their specifications in terms of temporal stability (magnitude and phase) and reproducibility are certainly not as well characterized as the clinical systems from the major vendors. In addition, features such as sophisticated eddy current characterization and compensation, feedback control of RF and gradient systems and continuous performance monitoring are either not present or must be designed from scratch. Although the lack of such features may not manifest themselves in standard low-field imaging, they do become critical when data processing algorithms such as in MR fingerprinting, which depend on fitting routines which assume perfect (or perfectly characterized) performance, are used. As an example, in our early work [45] we already found that our *T*_2_ values were significantly longer than those measured by conventional multi-echo fully sampled sequences.

We have performed numerous experiments as an attempt to understand the discrepancy in our study. We determined that one factor was the particular transmit–receive switch used, which in low-field systems is usually a simple lumped element quarter-wavelength equivalent with passive diodes, unlike the more sophisticated designs on conventional clinical systems. This caused the low tip angles used at various points in the fingerprinting sequence actually having a much lower tip angle than programmed. When the transmit/receive switch was replaced, the T_2_ values became much closer to the “gold standard.” A second factor, related to the SNR dependence of the fitting routine, was that most fingerprinting trains on clinical systems use a maximum tip angle of 60° due to SAR considerations. However, low field has no such restrictions, and by increasing this angle to 90° we were able to increase the contribution of stimulated echoes generated along the MRF train, which also resulted in improved fitting, as predicted by simulations (see Online Resource 6). However, we have not performed optimizations with respect to this parameter, and so it is not yet clear what effects it would have on the accuracy of T_1_ and T_2_ quantification. Higher tip angles could potentially further increase the T_2_ sensitivity, but this needs to be further investigated. A third factor was to include the effects of the temperature-induced changes in resonant frequency into the data processing (see Online Resource 7): these are particularly important to account for in vivo scanning where shifts of several hundreds of Hertz may occur during the scan. Using higher tip angles, improved hardware and accounting for field drift have given us at best percentage errors (with respect to spectroscopy values) below 8% for *T*_1_ and below 6% for *T*_2_ in phantoms, with repeatability tests showing a maximum difference of 11% for *T*_1_ and 26% for *T*_2_. However, the sensitivity to environmental changes and small changes in the parameter settings seems to be high, and therefore, long-term reproducibility needs to be better characterized in the future. This also requires the design of more robust, tailored MRF encoding approaches that better meet existing hardware constraints.

We showed that the effect of undersampling on the matched relaxation times was negligible using MC as reconstruction scheme. This effect could, however, be stronger when the center of k-space is not representative for the outer k-space region in terms of low-rank structure. This could be the case for example when *B*_0_ effects are stronger, since they introduce phase changes for higher frequencies, or when gradient nonlinearities become worse for high gradient strengths. This is also depending on the order used in the sampling scheme. We used an ordered, but incoherent, sampling scheme in which the jumps between successive k-space lines in one MRF train are small, such that also eddy-current effects are minimized.

The MRF sequence used in this work was optimized for differentiating between the relaxation times expected in the phantom at low field. The final sequence shows a relatively large number of ‘peaks’ in the train compared to those used at high field [46, 47]. This could be an effect of the shorter relaxation times compared to high field, or of the low flip angle number constraint, which was motivated by these short relaxation times. Since high-field MRF approaches typically use 500 to 1500 flip angles in the MRF train to achieve sufficient *T*_2_ encoding, it is worth investigating whether a longer (optimized) MRF train can reduce the standard deviation and improve the accuracy of the *T*_2_ measurements in this low-SNR regime.

The parameter maps obtained with MRF contained more noise compared to those obtained with reference techniques (IR and MESE), resulting in a larger standard deviation of the relaxation times. This can in part be explained by the fact that MRF data were acquired with an undersampling factor of 8.6, as opposed to fully sampled acquisitions for the reference techniques. The long scan time for the fully sampled reference techniques limited the through-plane thickness of the reference data to 8.3 mm, which is much larger than the 3.0 mm partition thickness for MRF. This could also explain the difference in level of observable structure between the MRF *T*_1_ maps and the reference *T*_1_ maps. An undersampling factor larger than 5 would need to be implemented for the reference scans to make the combined acquisition more time-efficient than the MRF scans, and such large undersampling for a temporal dimension of size 6 (TI) and 10 (TE) would likely lead to too much loss of information in a potential model-based reconstruction process. Online Resource 5 shows that a comparable through plane resolution for MRF and reference data (by retrospective averaging the MRF data in partition direction image-space) would reduce the noise difference between the parameter maps considerably. The amount of noise in the reference parameter maps is, however, still lower due to the inherent difference in SNR between spin echo and spoiled gradient echo-based acquisitions. The spoiled SSFP sequence distributes the magnetization among the higher-order coherence pathways, which do not directly contribute to the sampled echoes, and is therefore not optimal in terms of SNR. Balanced SSFP sequences would result in significant banding artifacts and complete signal loss in areas of strong B_0_ inhomogeneities and would therefore not be suitable in this case. Such effects are already observed to some extent with the spoiled SSFP sequence (see Fig. 7), which is known to be much less sensitive to inhomogeneous B_0_ fields. Although the MRF sequence shows a lower SNR, it samples M_0_, *T*_1_ and *T*_2_ maps more efficiently and currently at a higher resolution than its conventional quantitative mapping counterparts. This might make MRF interesting as an efficient way of producing multiple “synthetic contrasts” from a single scan as outlined in Online Resource 4.

The strong B_0_ inhomogeneities away from the center of the bore resulted in image distortions in the MRF images as well as in the matched parameter maps. These effects were corrected by incorporating an MRF-estimated ΔB_0_ map in a model-based reconstruction [39]. The maximal difference of 105 Hz between the MRF and TSE-based B_0_ map was potentially introduced by the difference in acquisition sequences used (gradient-echo vs spin-echo) or a temperature drift during and in between either of the scans. The remaining reduced signal area at the left side of the calf in Online Resource 3 is likely a result of (through-plane) intra-voxel dephasing, as shown also in Fig. 7, for which the current model-based reconstruction algorithm does not correct. Future work could correct for in-plane and through-plane dephasing by incorporating a higher resolution ΔB_0_ map into the reconstruction model, either by directly reconstructing the images on a higher resolution, or by including multiple ΔB_0_ frequencies for each voxel in the encoding matrix [48, 49]. It should also be further investigated whether linear field drift correction, as performed in Online Resource 7, improves the accuracy of the MRF parameter maps sufficiently in all in vivo scans, where field drift may not always be approximately linear.

In conclusion, volumetric MRF parameter maps using a spoiled SSFP sequence have been acquired on a 50 mT permanent magnet system in a scan time of 13 min. While the effective resolution of the acquired maps (~ 2.5 mm) is reduced compared to conventional data acquisition methods (IR/MESE) at low field and compared to MRF at high field, sufficient resolution to delineate major anatomical structures is still achieved. The MRF parameter maps were slightly overestimated with respect to T_1_, but severely overestimated with respect to T_2_. Improved hardware components (transmit-receive switch), the use of higher maximum flip angles or other MRF encoding approaches, and field drift correction can be impacting factors. Further work will help to understand and to control the remaining discrepancy and the reproducibility challenge, which are needed for MRF to become a practical operating sequence on very low-field point-of-care MRI systems in the future.

## Supplementary Information

Below is the link to the electronic supplementary material.

Supplementary file1 (PDF 719 KB)

## Data Availability

The data that support the findings of this study are available on request from the corresponding author. The data are not publicly available due to privacy or ethical restrictions.
