# Supplementary material for: 3D magnetic resonance fingerprinting on a low-field 50 mT point-of-care system prototype: evaluation of muscle and lipid relaxation time mapping and comparison with standard techniques
Source: MAGMA. 2023 May 18;36(3):499–512. doi: 10.1007/s10334-023-01092-0 (PMC10386962; doi:10.1007/s10334-023-01092-0)

Online Resources

Online Resource 1. The effect of undersampling on the reconstructed images and the matched relaxation times. Before MC reconstruction, large undersampling artifacts are present in the MRF images. After MC, the image quality is close to that of the fully sampled MRF images. The difference in matched relaxation times is negligible. The error bars represent standard deviations computed in each of the ROIs.

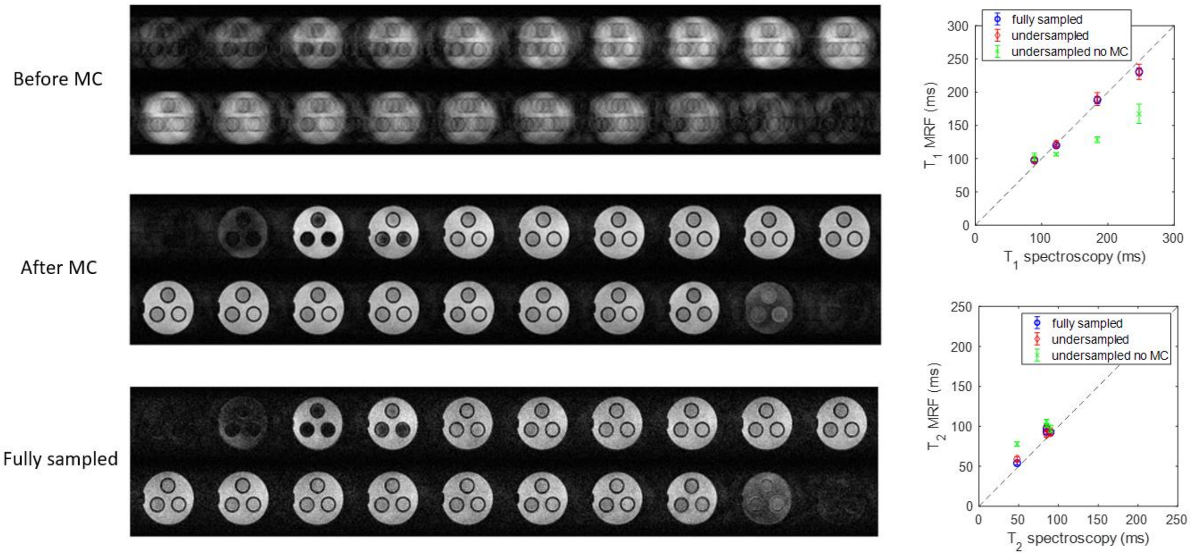

Online Resource 2. Volumetric relaxation time maps in one healthy volunteer before excitation profile correction. The Sinc-shaped excitation profile results in higher T_1 (A) and T_2 (B) values and more noise towards the edges of the excited region. The M_0 maps (C) show lower values towards the edges due to a reduced B_1^+ and receive coil sensitivity in these regions.

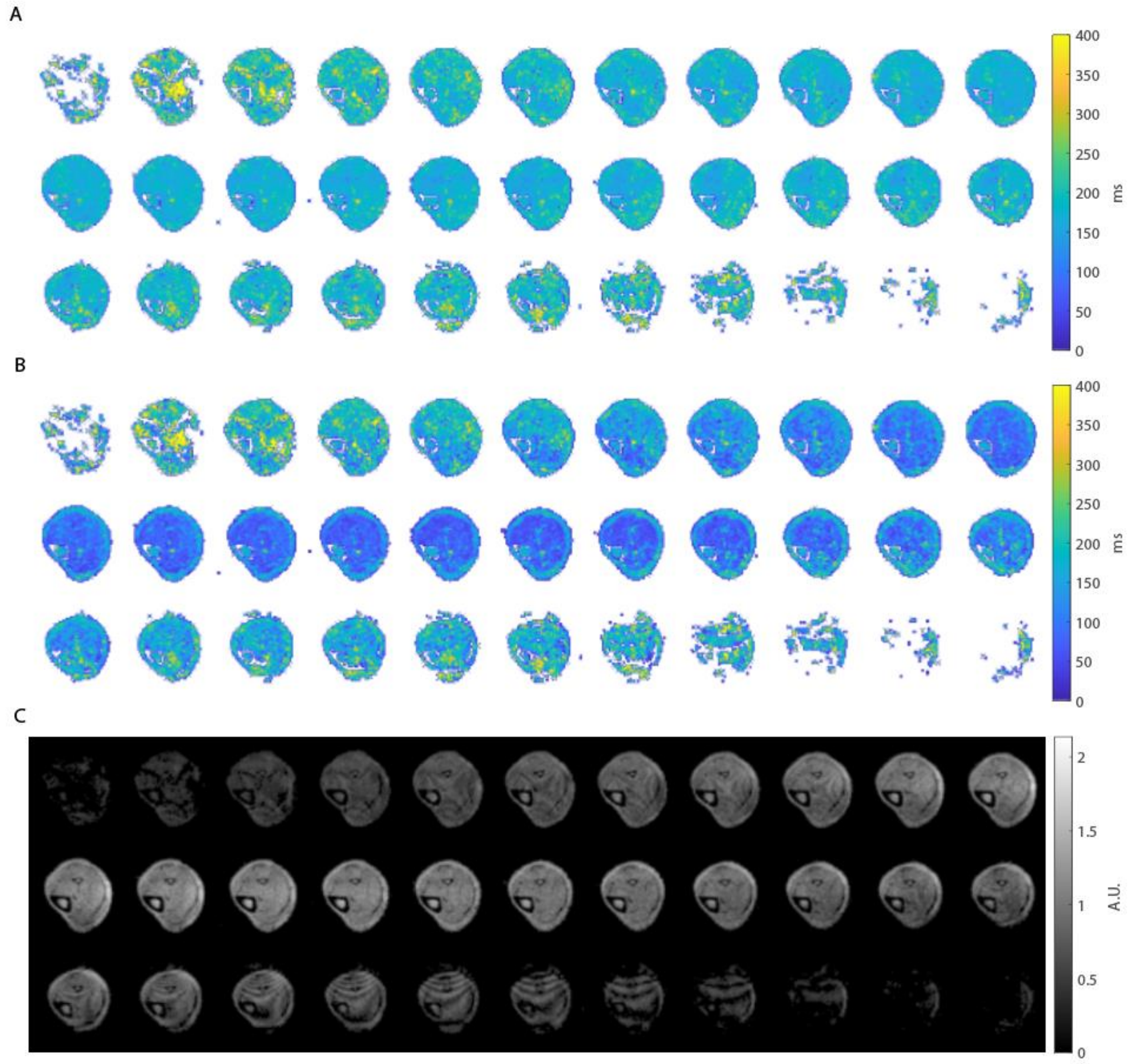

Online Resource 3. MRF with integrated B_0 estimation and distortion correction of the final MRF images. (A) Using an alternating TE pattern (TE_1 = 6 ms, TE_2 = 6.15 ms) along the MRF train instead of using a constant TE allows B_0 estimation from the MRF data. (B) Schematic overview of the processing pipeline. The grey boxes represent the extra processing steps compared to the standard processing pipeline. The MRF images were first split in two image series, one corresponding to TE_1 and one to TE_2. These two image series were reconstructed with matrix completion to obtain full k-space data. Second, an averaged image was computed from the image series for each of the TEs, resulting in two images with different B_0-induced phase. A B_0 map was computed from these images using total variation regularization. The B_0 map was used to correct the image distortions using a model-based reconstruction for each of the MRF images. The corrected MRF series are matched to the simulated dictionary. Note that this approach does not require any alterations to the dictionary simulation. (C) Using an alternating TE pattern along the MRF train (B_0-MRF) results in T_1, T_2 and M_0 maps of very similar quality to the those obtained with a constant TE (standard MRF). A B_0 map can be estimated from the alternating TE pattern, and be used in a model-based reconstruction to correct for B_0-induced image distortions (B_0-MRF corrected), especially in the area of the arrow. The remaining area with reduced signal, both present in the uncorrected and the corrected M_0 maps, is caused by (through-plane) intra-voxel dephasing which was ignored in the model-based reconstruction. Note that the model-based reconstruction may alter the noise appearance in the M_0 maps. (D) The B_0 map, estimated from the alternating TE pattern, is close to that of the B_0 map acquired with a TSE sequence, showing a maximum difference of 105 Hz. This difference is in the order of the accuracy (~10%) expected (39) and may be introduced by field drift or differences in eddy current effects between the MRF and the TSE sequences.

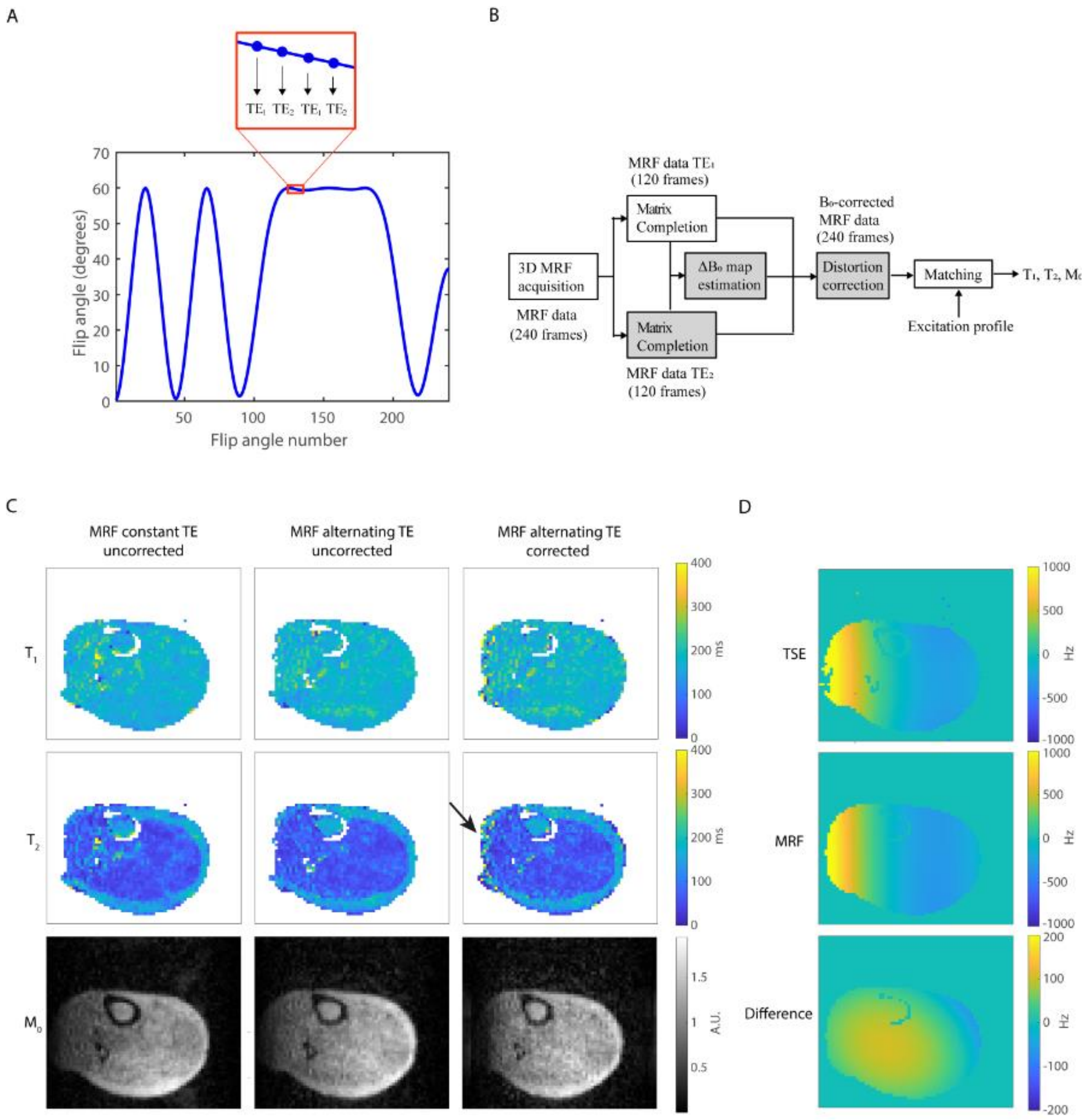

A
TE1 TE2 TE1 TE2
Flip angle (degrees)
Flip angle number
B
MRF data TE1 (120 frames)
Matrix Completion
B0-corrected MRF data (240 frames)
3D MRF acquisition
MRF data (240 frames)
ΔB0 map estimation
Distortion correction
Matching
T1, T2, M0
Excitation profile
Matrix Completion
MRF data TE2 (120 frames)
C
MRF constant TE uncorrected
MRF alternating TE uncorrected
MRF alternating TE corrected
T1
T2
M0
ms
A.U.
D
TSE
MRF
Difference
Hz

Online Resource 4. Synthetic MRI images of a volunteer's lower leg obtained with MRF and with reference relaxation time maps compared with the source reference data. MRF data were first averaged over three neighbouring slices before matching them to the dictionary to obtain relaxation time maps with a similar voxel size (2.5×2.5×9.0 mm^3) as the reference maps (2.5×2.5×8.3 mm^3). (A) M_0 and T_2 maps measured with MESE and with MRF were used to simulate TSE images at varying echo times TE=20 ms to TE=200 ms. (B) M_0 and T_1 maps measured with IR and with MRF were used to simulate IR TSE images at varying inversion times TI = 25 ms to TI = 300 ms. The overestimation of the MRF relaxation time values can be observed in the synthetic MRF image series, showing a slower signal decay than the synthetic MESE/IR image series. The finer structures between muscles are not visible in the synthetic MRF images, although visible in the synthetic MESE images, potentially due to the difference in SNR of the source data.

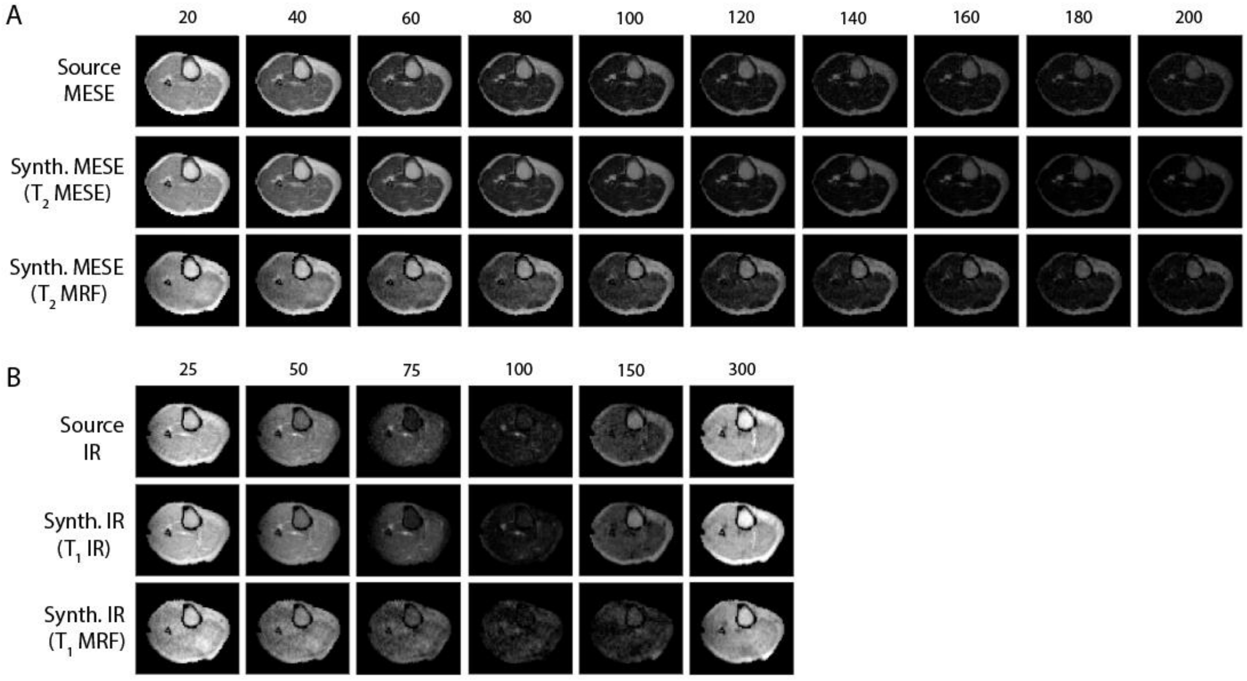

Online Resource 5. The effect of through-plane resolution on the amount of noise in the parameter maps in one volunteer's lower leg. Averaging the MRF source images (3.0 mm partition thickness) over three neighbouring slices in image space (~9 mm partition thickness), and performing the matching step afterwards, resulted in reduced noise in the MRF parameter maps. The SNR of the parameter maps obtained with the reference techniques (8.3 mm partition thickness) is still higher due to the inherently higher SNR of a spin-echo sequence compared to a spoiled gradient echo sequence, and due to the large undersampling (R=8.5) performed in the MRF experiments.

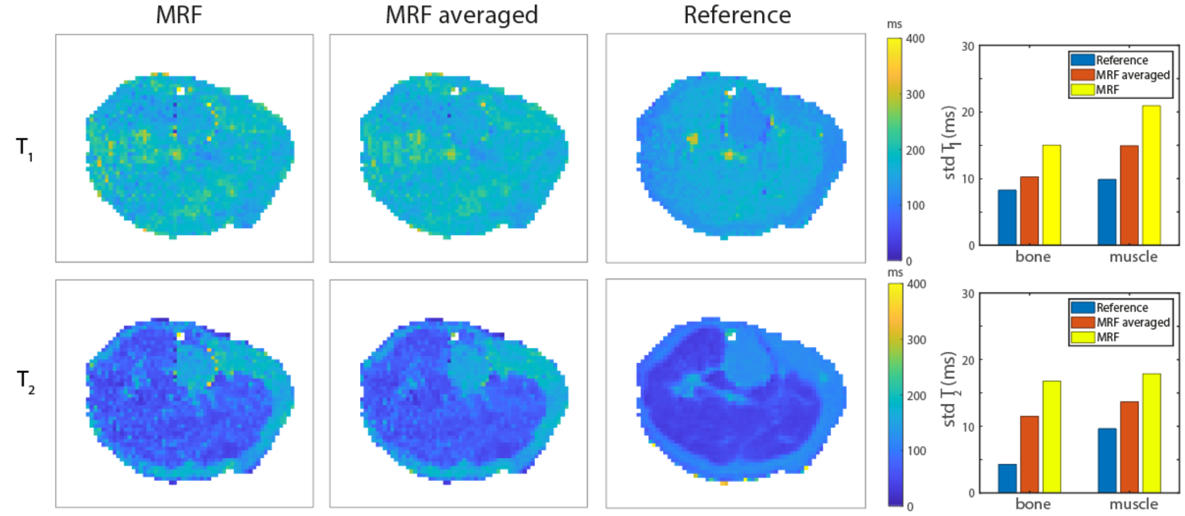

Online Resource 6. Sensitivity of T_2 to the maximum flip angle in the MRF sequence. Using a maximum flip angle of 90 degrees substantially improves the accuracy of the matched T_2 values in the phantom compared to using a maximum flip angle of 60 degrees. This can be explained by the fact that larger flip angles generate higher stimulated echo signals and therefore increase the T_2 encoding capability, as was confirmed by simulations. Note that for these low field systems, increasing the maximum flip angle in the MRF sequence is not limited by SAR. Repeated experiments on another day show noticeable variations in matched parameters, suggesting that the reproducibility of the approach needs to be further improved. The error bars represent standard deviations computed in each of the ROIs.

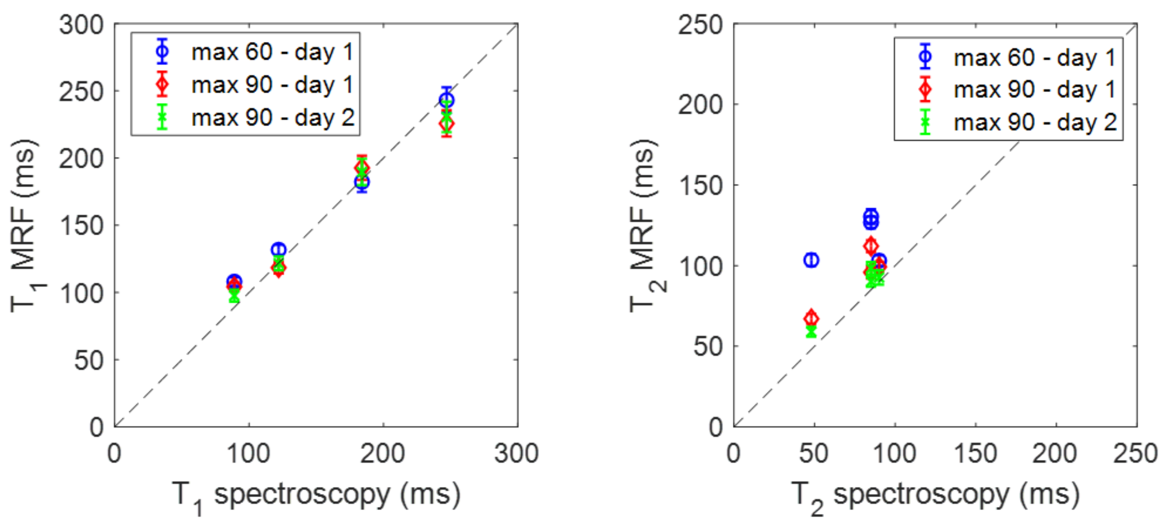

Online Resource 7. The effect of field drift correction. A phase correction was applied in k-space for each of the acquired lines before MC reconstruction. This assumed a linear field drift over the duration of the scan, tracked by f0 measurements right before and after the MRF scan as simple field navigators. The effect of the field drift on the matched parameter maps is most pronounced for the fully sampled scans, which took much longer to acquire than the undersampled scans. The correction is able to eliminate most of this effect, bringing the relaxation times close to the reference values. The error bars represent standard deviations computed in each of the ROIs.

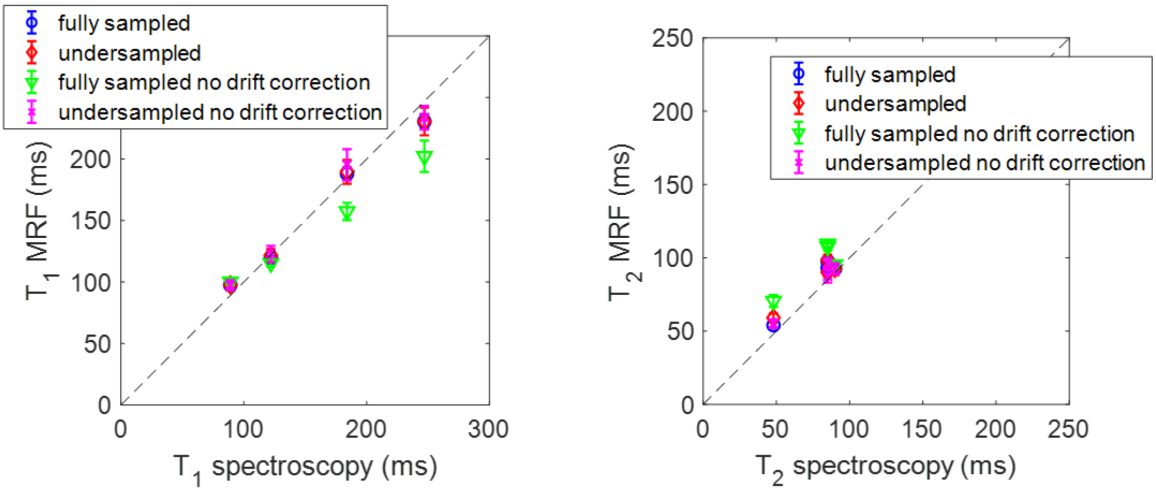

Supplement: Supplementary file 1 — Supplementary file1 (PDF 719 KB) [file 10334_2023_1092_MOESM1_ESM.pdf]
